# Anterior Shoulder Dislocation Complicated by Hill-Sachs Lesion

**DOI:** 10.7759/cureus.16925

**Published:** 2021-08-05

**Authors:** Nofel Iftikhar, Thor S Stead, Latha Ganti, Ilya Aleksandrovskiy, Frank Fraunfelter

**Affiliations:** 1 Emergency Medicine, Trinity Preparatory School, Winter Park, USA; 2 Medicine, Warren Alpert Medical School, Providence, USA; 3 Emergency Medicine, Envision Physician Services, Plantation, USA; 4 Emergency Medicine, University of Central Florida College of Medicine, Orlando, USA; 5 Emergency Medicine, Ocala Regional Medical Center, Ocala, USA; 6 Emergency Medicine, HCA Healthcare Graduate Medical Education Consortium Emergency Medicine Residency Program of Greater Orlando, Olrando, USA

**Keywords:** anterior dislocation, shoulder dislocation, humeral head, closed reduction, hill-sachs lesion

## Abstract

We present the case of a 60-year-old woman with a Hill-Sachs lesion caused by an anterior shoulder dislocation (SD) as a result of her falling on her left shoulder at a local restaurant. While the diagnosis of an anterior SD is commonplace, the involvement of a Hill-Sachs lesion can complicate the treatment needed to manage the SD. Crucial aspects of treatment include performing closed reduction followed by acute immobilization in a timely manner and prompt consultation with orthopedic surgery.

## Introduction

Shoulder dislocations (SDs) are a relatively common complaint in the Emergency Department (ED). Fifty percent of all reported joint dislocations are classified as SDs [1]. SDs are divided into two categories, anterior and posterior, with anteriorly dislocated shoulders accounting for the majority (95%-97%) of cases [2]. Anterior SDs are identified by the displacement of the head of the humerus bone in front of the glenoid [3-5]. A Hill-Sachs lesion is a compression injury in the humeral head, which is often caused by the dislocation of the glenoid rim such as during an anterior SD [6]. Because of this, Hill-Sachs lesions are primarily discovered during the x-rays obtained in identifying an anterior SD [7]. The authors describe a case of a Hill-Sachs lesion coupled with an anterior SD and the treatment provided.

## Case presentation

**Figure 1:**
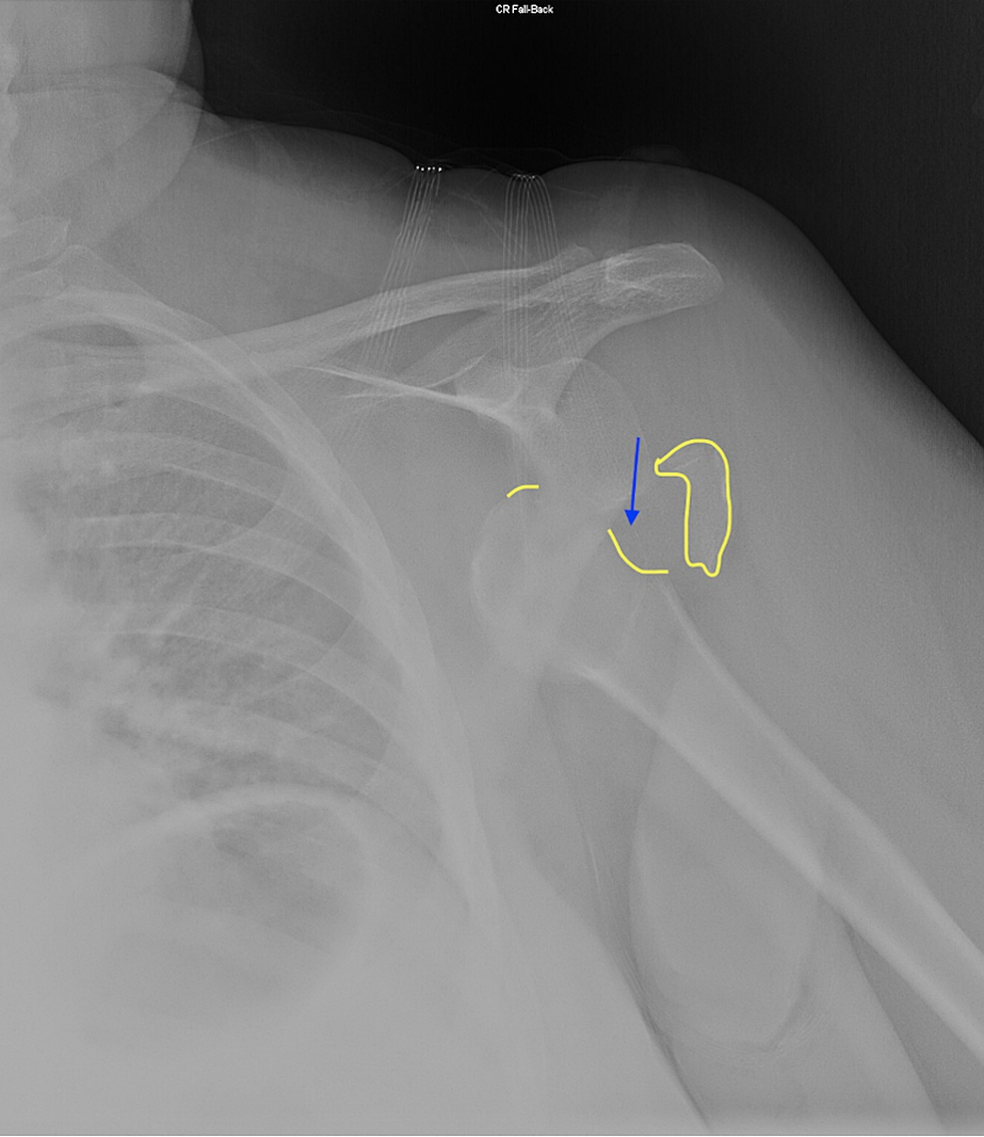
Patient’s shoulder radiograph pre-reduction with an outline of the Hill-Sachs fracture and an arrow pointing toward the Hill-Sachs lesion on the humeral head. Outlined in yellow is a loose fracture fragment.

A 60-year-old woman complaining of severe pain in her left shoulder presented to the ED. The patient had fallen onto her shoulder at a local restaurant. She did not hit her head in the fall but did hit the tip of her nose. Her nose was bruised, but she did not have any difficulty breathing through it. The patient's vitals were as follows: O_2_ saturation 97% on room air, blood pressure 190/101 mmHg, mean arterial pressure 130 mmHg, temperature 97.4°F, pulse 87 beats per minute, and respiratory rate 16 breaths per minute. Throughout the examination, the patient was alert and answering questions but was clearly in pain. The left arm was slightly abducted and externally rotated. There was an apparent defect when compared to the right shoulder. The patient had good radial, brachioradialis, and axillary pulses. The skin was warm and dry. Although an anterior SD was evident on clinical examination, the patient was sent to radiology for an x-ray to look for any associated fractures. The x-ray featured a clearly anteriorly dislocated shoulder associated with a Hill-Sachs lesion on the humeral head (Figure 1).

**Figure 2:**
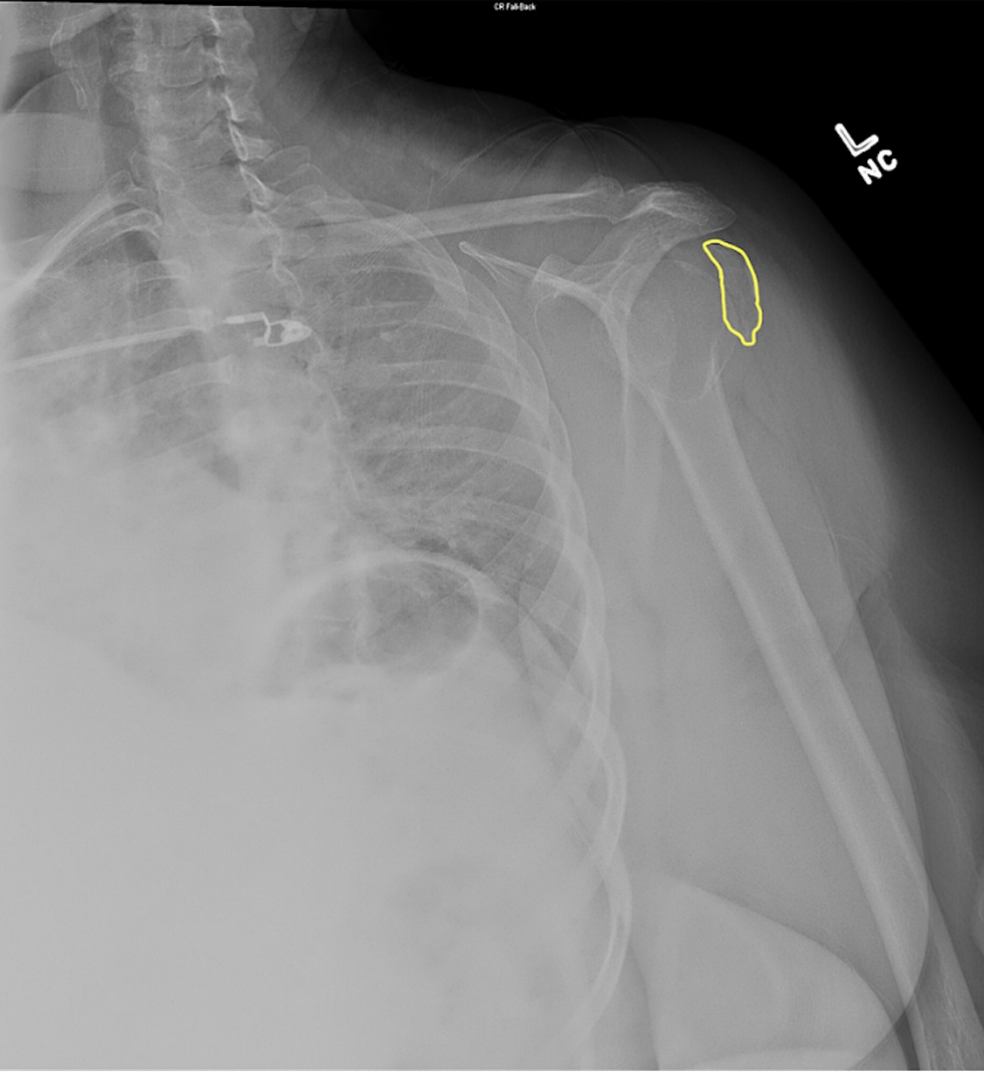
Patient’s shoulder radiograph post-reduction with an outline of the Hill-Sachs fracture.

Orthopedic surgery was consulted and recommended closed reduction in the ED with orthopedic office follow-up. Following informed consent, the patient underwent conscious sedation with intravenous ketamine and etomidate. The patient tolerated the procedure well, and the procedure was a success. The post-reduction x-rays depict regular alignment between the humeral head and the glenoid (Figure 2).

Post-reduction, the patient could fully range her shoulder and there was no longer a void in the shoulder capsule. Sensation over the deltoid was intact. The patient's vital signs following the reduction were O_2_ saturation 99% on room air, blood pressure 148/97mmHg (this slightly elevated blood pressure is easily explained by the patient's pre-existing hypertension), mean arterial pressure 114 mmHg, temperature 98.4°F, pulse 88 beats per minute, and respiratory rate 16 breaths per minute. The patient’s arm was placed in a sling and the patient was discharged to follow up with orthopedics.

## Discussion

**Figure 3:**
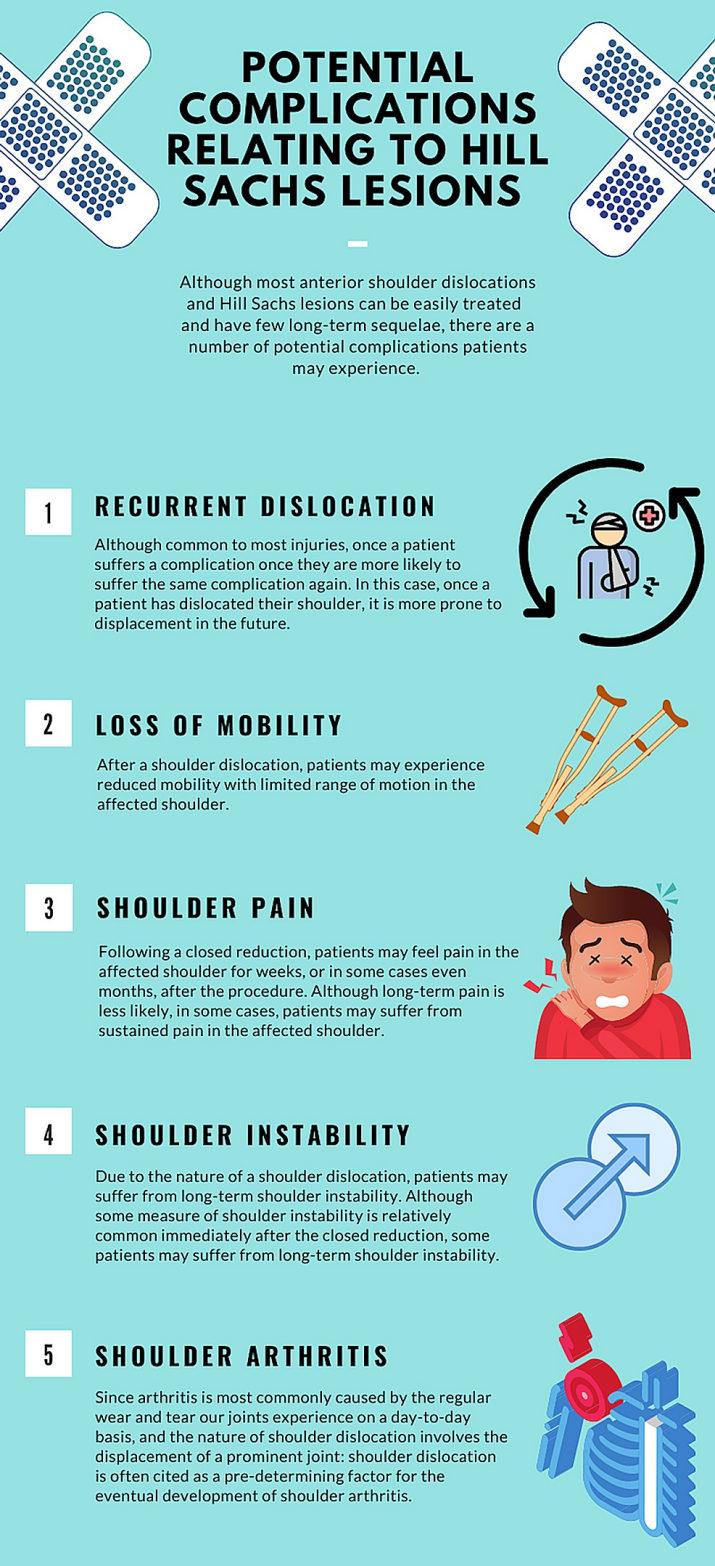
Infographic summarizing potential long-term complications associated with Hill-Sachs lesion.

Anterior SDs are primarily caused by forced abduction, external rotation, and hyperextension, all of which are expected consequences of patients falling on their outstretched arm or their shoulder, injuries sustained in sports, or trauma (Figure 3) [8].

Symptoms of anterior SD include severe pain, numbness, deformity, and swelling. X-ray imaging is sufficient to identify most anterior SDs and any associated fractures. Diagnosis can also be made at the bedside more swiftly with the point of care ultrasound (POCUS). POCUS is slowly gaining traction in the medical community, especially in the ED, as it is seen as more convenient (saving time), less expensive, non-invasive, and does not involve radiation. Although in the past, shoulder radiography was seen as the preferred method of diagnosing SDs, in the present day, the sensitivity and specificity of POCUS in identifying SDs is 100%. Much like the diagnosis of an SD via a shoulder radiograph, POCUS also features a picture of the shoulder joint with the humeral head clearly misaligned relative to the glenoid [9-12]. An anterior SD is identified by a humeral head that is “deeper” than the adjacent glenoid fossa.

Standard treatment consists of closed reduction followed by an extended period of immobilization to allow for complete healing [3]. However, the standard of care can vary for anterior SDs complicated by a Hill-Sachs lesion. Treatment of anterior SDs with accompanying Hill-Sachs lesions is primarily dependent on the size of the lesion on the head of the humerus. If the lesion occupies less than 20% of the head of the humerus, the standard treatment of immobilization and physical therapy can be executed. If it occupies more than 20% of the head of the humerus; however, treatment options include bone augmentation, remplissage, disimpaction, and limited resurfacing [13]. The process of bone augmentation is primarily divided into two procedures: glenoid augmentation and humeral head augmentation. Glenoid augmentations involve the lengthening of the articular arc of the glenoid via allograft tissues, and humeral head augmentations involve the filling of the Hill-Sachs lesion to restore regular anatomy. Another procedure, remplissage, is meant to convert an intra-articular defect into an extra-articular defect using soft tissue to prevent engagement of the Hill-Sachs lesion. Disimpaction, another treatment for Hill-Sachs, is focused on restoring the normal anatomy of the humeral head by elevating the fracture itself and reinforcing it with a bone graft. Limited resurfacing, much like glenoid bone augmentation, defines the procedure of installing a metal implant in hopes of restoring the humeral head articular arc. Although there are a number of procedures orthopedic surgeons use to treat Hill-Sachs deformities, the ones mentioned above are the most commonly performed [13-16]. Most patients will not suffer complications. However, possible complications related to the Hill-Sachs lesions are discussed in Figure 3 [4,17]. 

Another way to categorize the size of Hill-Sachs lesions is based on depth. The depth of the lesion reflects the amount of damage to the opposite side of the joint - the anterior capsule and the labrum [18]. If depth is less than 1/8, then closed reduction of the SD is appropriate and no specific treatment for the Hill-Sachs lesion is necessary. If the depth is between 1/8 and 1/4, remplissage is typically performed. Lesions larger than 1/4 are rare and are typically treated with disimpaction, where the defect is filled in with bone or sometimes a metal implant [19].

## Conclusions

Anterior SDs can sometimes be complicated by the co-occurrence of a Hill-Sachs lesion. Small lesions can be managed with routine closed reduction, shoulder sling, and orthopedic follow-up. Larger lesions may need further therapy. Some patients will have long-term complications related to the Hill-Sachs lesion.
